# Integrating social–emotional learning into community nursing curriculum: a quasi-experimental study on enhancing emotional competence and professional readiness in nursing students

**DOI:** 10.3389/fmed.2026.1837342

**Published:** 2026-06-22

**Authors:** Jing Cheng, Qi Cheng, Yingting Wu, Li Huang, Yuehong Wu

**Affiliations:** 1 School of Nursing, Anhui University of Chinese Medicine, Hefei, Anhui, China; 2 Laboratory of Geriatric Nursing and Health, Anhui University of Chinese Medicine, Hefei, Anhui, China

**Keywords:** community nursing, emotional competence, nursing students, quasi-experimental study, social–emotional learning

## Abstract

**Objective:**

To evaluate the potential impact of a Social–Emotional Learning (SEL)-integrated pedagogical intervention in an undergraduate *Community Nursing* course on enhancing students’ socio-emotional competencies and professional readiness.

**Methods:**

A quasi-experimental design was employed. Two intact classes of nursing students were assigned to an experimental group (*n* = 56) receiving SEL-integrated instruction or a control group (*n* = 54) receiving traditional lecture-based teaching. The 12-week intervention for the experimental group was built around the five core SEL competencies, utilizing a blended online-offline flipped classroom model featuring scenario simulations, structured reflection, and faculty training, aligned with Kolb’s experiential learning cycle. Outcomes were measured post-intervention using standardized scales assessing emotional experience (PANAS), emotion regulation strategies (ERQ), empathy (JSE-HPS), attitudes toward communication skills learning (CSAS), and ethical decision-making (JAND).

**Results:**

Compared to the control group, students in the experimental group demonstrated significantly higher levels of positive affect, greater use of cognitive reappraisal, enhanced empathy across all dimensions, more positive attitudes toward learning communication skills, and stronger ethical decision-making abilities. They also reported significantly lower levels of negative affect and less reliance on expressive suppression (all *p* < 0.001, with moderate to large effect sizes, which were especially large for emotional experience dimensions).

**Conclusion:**

The results indicate that the efficacy of SEL-integrated instruction lies in its mechanisms, such as contextualized simulation, structured reflection, and supportive feedback, which foster the transition of socio-emotional competencies from explicit learning to internalized application. This approach may address a training gap and potentially enhance preparedness for the relational demands of community practice.

## 1 Introduction

Nursing education serves as a critical component in the training system for healthcare professionals, with its quality directly shaping the future standards of medical services and patient health outcomes. As an interdisciplinary field integrating public health and nursing theories, community nursing focuses on applying the nursing process to deliver continuous, dynamic, and professional services aimed at health promotion, disease prevention, health maintenance, and alleviation of suffering for individuals, families, and entire populations within the community (1). It represents not only a vital branch of nursing science, but also a crucial link connecting clinical individual care with public health population-based interventions. In cultivating students’ comprehensive competencies for community-oriented and primary care settings, community nursing plays an irreplaceable role (2).

Despite its crucial role, nursing education faces evolving challenges in preparing students for these complex community settings. Within the rapidly evolving technological landscape of healthcare, current undergraduate nursing education has, to some extent, revealed a systematic gap in cultivating socio-emotional competencies. Issues such as insufficient empathy, weak communication skills, difficulties in navigating complex interpersonal situations, and an increased early risk of professional burnout among students have drawn scholarly attention (3). This gap, if not addressed proactively, may not only constrain their capacity to deliver high-quality, humanistic care but could also exacerbate workforce attrition due to an inability to effectively manage work-related stress and emotional demands (4).

To address the aforementioned challenges, the systematic educational paradigm of Social–Emotional Learning (SEL) offers a critical theoretical perspective and practical framework (5, 6). Rooted in social learning theory, SEL emphasizes that individuals learn and develop through observation, imitation, and authentic social interaction. It aims, via structured educational processes, to systematically cultivate learners’ core competencies in understanding and managing their own emotions, establishing and maintaining positive relationships, and making responsible decisions. These competencies are primarily organized into five core dimensions: self-awareness, self-management, social awareness, relationship skills, and responsible decision-making (7). Research indicates that SEL plays a significant role in fostering empathy and resilience among healthcare professionals (8, 9). Empathy enables them to better understand patient perspectives and emotions, while resilience aids in more effectively coping with workplace challenges and stress, which is crucial for professionals often operating in high-pressure environments (5, 7, 10, 11).

Given this context, the systematic integration of SEL into the instruction of *Community Nursing* appears both particularly necessary and urgent. The working environment of community nursing is characterized by high complexity and uncertainty, requiring nurses to engage deeply within diverse sociocultural settings and build trusting relationships with clients from varied backgrounds. The effectiveness of their work relies not only on proficient clinical skills but also, to a significant extent, on a deep capacity for humanistic care, acute socio-emotional awareness, and robust psychological resilience. Specifically, community nurses require sound abilities in emotional awareness and regulation (self-awareness and self-management) to cope with the emotional demands of their work (12); they must deeply understand and respond to the circumstances and feelings of their clients (social awareness/empathy) to establish trust (13); they need to employ effective communication skills to collaborate with clients and their families (relationship skills/communication competence) (14); and they must also make ethically sound professional decisions within complex community contexts (responsible decision-making) (15). However, traditional teaching models that primarily focus on knowledge and skill transfer often prove inadequate in systematically cultivating such contextualized, affective, and relational core competencies. Therefore, introducing the SEL framework and deeply integrating it with the practical realities of community nursing represents an essential step to address current educational gaps and to align with the necessary shift in nursing education towards a model that balances technical proficiency with humanistic competencies.

The primary objective of this study was to explore, based on curriculum design principles for SEL integration and through a quasi-experimental design with a control group, the specific pathways and effectiveness of incorporating SEL into the *Community Nursing* curriculum. Focusing on the five core SEL competency dimensions, the research employed corresponding standardized measurement tools to systematically evaluate the outcomes of the curricular intervention. This aimed to provide scientific evidence and practical guidance for cultivating outstanding community nursing professionals who possess both the technical “hard skills” and the socio-emotional “soft skills” essential for excellence in the field.

## 2 Methods

### 2.1 Research design and participants

Following ethical approval from the Ethics Committee of Anhui University of Chinese Medicine (No. AHUCM-HSS-2025010), this study employed a quasi-experimental design, implementing a teaching intervention within a 12-week *Community Nursing* course. Following the principles of a quasi-experimental design, two intact classes from the same grade level of the undergraduate nursing program were selected via convenience sampling and designated as the experimental group (*n* = 56) and the control group (*n* = 54), respectively, to compare the effects of SEL-integrated instruction versus traditional teaching. The two groups were kept consistent in terms of total course hours, instructor qualifications, and assessment methods, differing only in teaching content and methodology, thereby effectively controlling for the interference of external variables.

### 2.2 Intervention for the experimental group

#### 2.2.1 Pedagogical philosophy and design framework

Instruction for the experimental group was structured around the framework of the five core SEL competencies: self-awareness, self-management, social awareness, relationship skills, and responsible decision-making. The pedagogical approach adopted a blended flipped classroom model, utilizing the Chaoxing Learning Platform (a widely used Chinese learning management system) to construct a teaching pathway of “online cognitive preparation – offline experiential internalization – online reflective extension.”

The course comprised a total of 48 credit hours: 24 h of theoretical instruction, 20 h allocated across five experiential learning sessions, and 4 h for self-directed study. The curriculum was designed using the backward design approach, an educational framework developed by Wiggins and McTighe that emphasizes starting with desired end goals. In this study, this process began by defining the terminal SEL competency objectives before planning instructional activities and assessments. Teaching activities and assessment methods were then developed in reverse to align with these objectives.

A core principle of the course design was to prioritize guided practice over extensive lecturing. Consequently, 20 credit hours, systematically embedded within the experiential learning sessions across various instructional units, were dedicated to specialized, hands-on activities. Table 1 details the alignment between core course modules, targeted SEL competencies, and exemplar teaching activities.

**Table 1:** Alignment of course modules with SEL competencies and exemplar teaching activities.

Course core module	Embedded SEL competencies	Exemplar teaching activity	Credit hours
Chronic disease management	Self-management: assisting patients with emotional regulation and behavioral change. Social awareness: understanding the long-term impact of chronic conditions on patients’ families and social functioning. Relationship skills: conducting motivational interviewing and shared decision-making. Responsible decision-making: formulating individualized care plans under resource constraints	Chronic disease self-management support group simulation: simulating community health workshops centered on common chronic diseases like diabetes and hypertension. Students role-play patients at different psychological stages or take on the nurse role, employing motivational interviewing techniques to foster patient self-management motivation. The group collaboratively discusses how to set feasible health goals and action plans within real-world constraints such as insurance policies and family support	4
Community rehabilitation nursing for persons with disabilities	Self-awareness: recognizing one’s own potential biases and emotional reactions towards disability. Social awareness: understanding the emotional needs and social challenges faced by persons with disabilities and their families. Relationship skills: applying empathy and supportive communication techniques	“perspective-taking” role-play: in groups, students simulate the communication barriers and social discrimination encountered by persons with disabilities during community rehabilitation. They practice empathetic responses from the nurse’s perspective, design personalized rehabilitation support plans, and simulate explaining these plans to family members.	4
Community geriatric nursing	Self-awareness: exploring personal attitudes and emotional experiences related to aging and functional decline. Social awareness: empathizing with older adults’ feelings of loneliness, loss, and need for dignity. Relationship skills: building trust with older adults and their family members	Life story interview and empathy practice: inviting community elderly volunteers or using standardized patients to portray individuals with early-stage dementia. Students, acting as interviewers, listen to their life stories, practicing active listening, emotional validation, and reminiscence therapy. They also simulate managing conflict situations (e.g., medication refusal, overprotective family members), guided to employ cognitive reappraisal strategies amidst emotional fluctuations	4
Community health nursing for women and children	Self-awareness: reflecting on personal potential stereotypes regarding gender roles and motherhood. Social awareness: appreciating the vulnerability and unique needs of women and children in healthcare. Relationship skills: using nonviolent communication to address sensitive topics	Sensitive topic communication simulation: scenarios such as difficulties with breastfeeding, vaccine hesitancy for children, or adolescent reproductive health counseling are designed for role-play. The focus is on practicing how to provide emotional support and scientific information with an open and accepting attitude while protecting privacy and respecting values. Observing groups then provide feedback based on the sel framework.	4
Family lifecycle health education	Social awareness: insight into the core health needs and cultural differences across various family developmental stages. Relationship skills: conducting family interviews and multi-member communication. Responsible decision-making: developing health education plans suited to the family’s actual circumstances.	Family lifecycle case study and discussion: selecting typical family cases (e.g., newlywed, child-rearing, empty-nest stages) for simulated home visits. Students identify potential health risks, analyze family interaction patterns, practice conveying health information within different cultural contexts, and ultimately formulate personalized health education prescriptions.	4

#### 2.2.2 Faculty training

This study represents the first implementation of the integrated SEL curriculum model by the participating faculty. To ensure that the SEL-integrated pedagogy translated effectively from conceptual design into high-quality classroom practice, a systematic, specialized training program was implemented for the course instructors. The training aimed to facilitate a threefold role transformation: from knowledge transmitter to learning facilitator, from skills demonstrator to emotional catalyst, and from classroom manager to creator of a psychologically safe environment.

*Training format*: A blended training model was implemented for six faculty members from the same college of nursing. The model included workshop seminars, online community co-learning, and classroom practice mentoring, delivered through three intensive face-to-face workshops and asynchronous discussions via the “Faculty Development” module on the Chaoxing app. All participants, whose teaching experience ranged from 10 to 20 years and who had no prior SEL training, engaged in training spanning the 2 weeks before the course began and the first 8 weeks of implementation. This design ensured reflective practice and application-based learning among faculty.

*Training content framework*: To ensure the effective translation of SEL principles into classroom practice, the training content was structured around four core instructional competencies required of faculty, designed as four progressive modules encompassing conceptual internalization, instructional facilitation, emotional support, and technological application.

*Module 1: internalization of SEL concepts and behavioral manifestations*: This module aimed to help instructors develop a deep understanding of the specific connotations of the five core SEL competencies within the context of community nursing education and translate them into observable, assessable classroom behavioral indicators. Training methods combined case analysis with behavioral profiling. Typical interpersonal interaction cases from community nursing practice were selected to guide instructors in identifying the SEL elements involved. Through group discussions, instructors collaboratively defined “specific student behaviors that demonstrate a given SEL competency during simulation activities,” fostering a unified, clear, and operational understanding of SEL objectives.

*Module 2: training in experiential teaching facilitation skills*: This module focused on the design, organization, and debriefing techniques for core SEL instructional activities such as role-plays and scenario simulations. It employed a training cycle aligned with Kolb’s experiential learning cycle (16): First, instructors participated in a complete SEL simulation activity as learners, experiencing the emotional dynamics and learning processes firsthand. Then, switching to the “facilitator” role, they practiced debriefing the same activity, rehearsing Socratic questioning techniques (e.g., “What emotions were you experiencing at that moment?” “What triggered that feeling?” “If you could do it again, at what point would you choose to adjust your response?”). Peer observation and immediate feedback helped instructors master the skills for guiding students toward deep emotional reflection.

*Module 3: classroom emotional support and crisis response*: Centered on an “emotional first-aid” protocol, this module aimed to enhance instructors’ ability to recognize, acknowledge, and appropriately manage student emotional fluctuations, thereby fostering a psychologically safe and ethically sound classroom environment. By reviewing video clips of actual classroom interactions, instructors were guided to identify key signals warranting intervention. Discussions focused on appropriate response strategies for different scenarios, ensuring the provision of sustained and professional emotional support.

*Module 4: application of online learning support tools and continuing professional development*: This module leveraged the “SEL Faculty Community” established on the Chaoxing app. Instructors learned to utilize the platform’s features, such as posting templates for structured reflection journals, organizing peer assessments among students, and using word cloud analytics to track students’ emotional trends, thereby providing technological support for online learning facilitation. Instructors were required to upload audio recordings of their own SEL classroom facilitation for peer review. Peers provided evidence-based feedback by referencing the specific, observable facilitation behaviors that were defined and agreed upon during the SEL competency training in Module 1. Online discussions addressed how to respond consistently and ethically to psychological distress revealed in students’ reflection journals, formulating unified ethical response guidelines to ensure the safety and standardization of online support. This online community fostered experience sharing and professional reflection among instructors, enhancing the overall training effectiveness.

#### 2.2.3 Online learning support

To scaffold the offline SEL experiential activities, the course utilized the Chaoxing app to construct an “SEL Affective Learning Community.” The specific functional designs were as follows:

*Pre-class affective priming (resource distribution)*: Prior to each offline SEL activity, instructors shared theme-related materials via the app to activate students’ “social awareness” and provide emotional context for the upcoming role-play.

*In-class affective documentation (interactive tools)*: During offline role-plays or simulations, instructors used the app’s “group task” function. Students in observer groups were tasked with using their mobile devices to document key interaction moments in real-time and provide immediate feedback on the role-players’ empathic responses using the “rating” function.

*Post-class reflection deepening (reflection journals)*: After each activity, students submitted structured reflection journals on the Chaoxing app. The journal was designed using an SEL-guided framework to prompt students to reflect on their emotional experiences, management strategies, empathy gains, and application plans (for the full journal template, see Section 2.2.4).

*Peer assessment and data archiving (process evaluation)*: Students conducted peer reviews of reflection journals on the app. Instructors utilized the app’s data analytics functions to track trends in students’ emotional expressions, providing evidence for targeted offline guidance.

#### 2.2.4 Instructional implementation

The experimental group utilized the 20 credit hours of practicum sessions for SEL-integrated experiential and reflective learning activities, distributed across the five core teaching modules (see Table 1). Implementation followed the blended instructional pathway of “online cognitive preparation – offline experiential internalization – online reflective extension.”

*Overall instructional implementation framework*: The implementation was student-centered, with each SEL-focused activity unfolding in four stages aligned with Kolb’s experiential learning cycle: Concrete Experience, Reflective Observation, Abstract Conceptualization, and Active Experimentation (16). Before class, students completed affective priming and cognitive preparation on the Chaoxing app. During class, competency internalization was achieved through high-engagement scenario simulations and structured discussions. After class, learning was deepened through online reflection and peer assessment. The specific implementation flow is detailed below.

*Online support: affective priming and cognitive preparation*: Three to 5 days before the offline activity for each module, instructors distributed relevant online learning resources via the Chaoxing app to activate students’ prior emotional experiences and prepare them for deep in-person engagement. This included: (1) Affective Awakening Materials: Sharing video clips, standardized patient communication audio, or authentic case texts related to the module theme. Students were required to complete a brief emotional diary after viewing, noting “the most moving moment” and “how I would feel if I were the community nurse.” (2) SEL Competency-Guiding Questions: Posting guiding questions in the app’s discussion forum to encourage preliminary thinking and peer exchange before class. (3) Contextual Background Knowledge: Distributing information on the community cultural backgrounds and family structure characteristics relevant to the module, helping students understand the health beliefs and behavioral patterns of different groups. Instructors monitored students’ preparatory emotional states via backend app data to dynamically adjust the focus of the upcoming offline activity.

*Offline experience: scenario simulation and structured reflection*: Offline sessions constituted the core for SEL competency internalization, primarily realized through three experiential learning strategies.

*① Scenario Simulation and Role-Play*: High-fidelity simulation activities were designed around emotionally challenging scenarios typical of community nursing practice. Taking the “Community Chronic Disease Care” module as an example, the process for a diabetes self-management support simulation was as follows: *Step 1: Scenario Introduction and Role Assignment (15 min)* – The instructor briefed the case background. Students were divided into “Nurse Group,” “Patient Group” (role-playing patients in denial, anxiety, and acceptance stages), “Family Group,” and “Observer Group.” *Step 2: Simulation Execution (30 min)* – Groups engaged in the scenario based on role cards. The Nurse Group employed motivational interviewing techniques to foster patient self-management, responding to potential emotional resistance. *Step 3: Role Rotation (20 min)* – Students switched roles for a second simulation to experience emotional responses from different perspectives. Throughout, Observer Group students used the app’s “group task” function to record key interaction points and effective communication behaviors in real-time, providing material for the subsequent debrief.

② *Structured Debriefing and Group Discussion*: The debriefing session immediately followed the simulation, representing a critical component of SEL instruction. Instructors facilitated a debriefing order prioritizing emotion. The “Patient Group” was first invited to share their feelings, followed by the “Nurse Group” explaining their emotional states and decision-making rationale. The “Observer Group” then provided constructive feedback based on the SEL framework. Finally, the instructor guided students in deeper reflection with questions like: “Were you aware of your own emotions at that time? What triggered them?” and “If you could do it again, at what moment would you pause and choose a different response?” This layered questioning helped students practice emotional awareness and cognitive reappraisal.

*③ Case Analysis and Ethical Deliberation*: Authentic, context-based cases involving value conflicts and ethical dilemmas in community nursing were introduced. Instructors guided students to identify the emotional drivers behind decisions, cultivating ethical sensitivity and multiple-perspective thinking. For instance, in the “Family Lifecycle Health Education” module, a case involving “empty-nest parents concealing their illness from their children” was used. Students engaged in ethical deliberation debates in small groups. Each group had to analyze the health needs, emotional appeals, and cultural values of different stakeholders from their respective perspectives before arriving at a responsible professional decision.

*Post-class extension: online reflection and peer assessment*: Within 24 h of the offline activity, students were required to log into the Chaoxing app to complete two tasks to consolidate learning: First, submit a structured reflection journal. The journal template, designed using an SEL-guided framework, contained five core questions: (1) What emotions did I experience during this simulation? What triggered them? (Self-Awareness); (2) How did I manage my emotions to maintain professional engagement? (Self-Management); (3) What new understanding did I gain about the patient’s/family’s situation by playing/observing others? (Social Awareness); (4) If I encounter a similar situation in future community practice, how will I apply today’s experience to make a more responsible decision? (Responsible Decision-Making). Second, participate in peer assessment. (5) Relationship Skills: Reflect on a key moment during the simulation or group debriefing where you actively practiced communication, collaboration, or conflict resolution. Each student was required to comment on the reflection journals of two randomly assigned peers from their group, providing specific feedback based on SEL competency demonstrations. Instructors used the app’s “word cloud analysis” feature to track keyword trends in students’ emotional expressions, identifying common confusions or emotional fluctuations to address in subsequent classes.

*Safeguard Mechanisms for Instructional Implementation*: To ensure the effective delivery of SEL activities, the research team established safeguard mechanisms. First, *Creation of a Psychologically Safe Environment*: Before each activity, instructors reiterated classroom principles of “non-judgment and experiential focus,” explicitly stating that emotional expression was acceptable and that any emotional reaction was a learning resource, not a problem. Second, *Emotional Emergency Response*: If a student exhibited strong emotional fluctuations due to deep role immersion, the instructor could pause the activity, offer brief one-on-one accompaniment and support, and, if necessary, suggest follow-up with the psychological counseling center.

### 2.3 Instructional scheme for the control group

The control group received instruction via the conventional *Community Nursing* curriculum, which did not incorporate the specialized SEL content. The teaching scheme followed the traditional lecture-based model, focusing on the same five core course modules as the experimental group to ensure consistent coverage of professional knowledge. The allocation of class hours was identical to that of the experimental group. However, classroom time was primarily devoted to theoretical exposition, knowledge assessment, and demonstration of standard nursing skills. The schedule for the community practicum was synchronized with the experimental group. Nonetheless, practicum tasks focused on technical operations and knowledge application, with post-practicum reports emphasizing factual description rather than guided emotional or ethical reflection. This design aimed to establish a baseline reflective of conventional teaching practices, enabling a clearer isolation of the net effect attributable to the SEL intervention.

### 2.4 Measurement instruments and data collection

To evaluate the intervention’s effects, five standardized measurement instruments were administered to students in both groups within 1 week after the course concluded:

#### 2.4.1 Positive and negative affect schedule (PANAS)

Developed by Watson and colleagues, the PANAS assesses an individual’s recent emotional experiences across two dimensions: positive affect and negative affect. It comprises 20 adjective items, with 10 items per dimension. Responses are recorded on a 5-point Likert scale (1 = *very slightly or not at all*, 5 = *extremely*). Separate total scores are calculated for positive and negative affect, with higher scores indicating more intense experiences of the respective emotion. The Chinese version of the PANAS has shown good psychometric properties, with a Cronbach’s *α* of 0.751 and a KMO of 0.847 (Bartlett’s test *p* < 0.001), supporting its structural validity for factor analysis (17).

#### 2.4.2 Emotion regulation questionnaire (ERQ)

Developed by Gross and colleagues, the ERQ measures an individual’s propensity to use two emotion regulation strategies: cognitive reappraisal and expressive suppression (18). The 10-item scale consists of a 6-item cognitive reappraisal subscale and a 4-item expressive suppression subscale. Items are rated on a 7-point Likert scale (1 = *strongly disagree*, 7 = *strongly agree*). Subscales are scored independently, with higher scores indicating a greater tendency to employ the corresponding strategy. The Chinese version of the ERQ has shown acceptable internal consistency (Cronbach’s *α* = 0.79) in prior research (19).

#### 2.4.3 Chinese version of the jefferson scale of empathy-health profession students (JSE-HPS)

Originally developed by Hojat and colleagues for health profession students, the JSE-HPS is designed to measure empathy within clinical and nursing contexts. The Chinese version encompasses three dimensions: perspective-taking, compassionate care, and standing in the patient’s shoes. It contains 20 items rated on a 7-point Likert scale (1 = *strongly disagree*, 7 = *strongly agree*). The total score ranges from 20 to 140, with higher scores indicating stronger empathic ability. This scale shows excellent reliability and validity in nursing student samples. In the present study, its Cronbach’s *α* coefficient was 0.86 (20).

#### 2.4.4 Communication skills attitude scale (CSAS)

Developed by Rees and colleagues, the CSAS evaluates medical and nursing students’ attitudes toward learning communication skills. It consists of two dimensions—positive attitudes and negative attitudes—with 13 items each, totaling 26 items. Responses are given on a 5-point Likert scale (1 = *strongly disagree*, 5 = *strongly agree*). Total scores for positive and negative attitudes are calculated separately, with higher scores reflecting a stronger inclination toward the respective attitude. The Chinese version of the CSAS has demonstrated excellent internal consistency, with a Cronbach’s *α* of 0.919 for the total scale and coefficients of 0.891 and 0.923 for its positive and negative subscales, respectively (21).

#### 2.4.5 Judgment about nursing decisions (JAND) questionnaire

The JAND scale was used to assess participants’ ethical decision-making ability. The instrument comprises two core dimensions: ethical choice and ethical action, covering a total of 48 items. Respondents rate each item on a Likert 5-point scale (1 = strongly disagree, 5 = strongly agree). The total score theoretically ranges from 76 to 380 points, with higher scores reflecting greater ethical decision-making competency. The Chinese version of the JAND has been validated in nursing education research, demonstrating acceptable internal consistency (*α* = 0.745) (22).

Research assistants blinded to group allocation administered and collected all questionnaires using a standardized, neutral script. They had no role in teaching, and students completed the surveys anonymously during class.

### 2.5 Data analysis

Statistical analyses were performed using SPSS 24.0. Normality was checked with the one-sample Kolmogorov–Smirnov test; variables meeting normality (*p* > 0.05) were compared via independent-samples *t*-tests, and those violating it were analyzed with the Mann–Whitney *U* test. The effect size for each comparison was calculated and reported to quantify the magnitude of observed differences. Cohen’s benchmarks were applied to classify effect sizes (small = 0.2, medium = 0.5, large = 0.8). All tests were two-tailed, with the significance level set at *α* = 0.05.

## 3 Results

### 3.1 Baseline characteristics of participants

We first compared the experimental and control groups on key demographic and educational background variables. The chi-square test revealed no significant difference in gender distribution (χ^2^ = 1.049, *p* = 0.306), and the Mann–Whitney *U* test showed no significant difference in age (Z = −0.394, *p* = 0.694). Furthermore, all participants had completed an identical core curriculum of basic medical theory and clinical introduction prior to the study. Quantitative comparisons confirmed no significant differences between groups in the proportion of students who had taken key prerequisite courses or in the total number of courses taken in the preceding semester.

### 3.2 Comparison of students’ emotional experiences

As shown in Table 2, independent-samples *t*-tests revealed significant differences between the experimental and control groups in both positive and negative affect following the intervention. Students in the experimental group reported significantly higher levels of positive affect and lower levels of negative affect compared to their counterparts in the control group, with large effect sizes for both dimensions.

**Table 2:** Comparison of PANAS scores between groups.

Dimension	Control group (*n* = 54)	Experimental group (*n* = 56)	*t*	*p*	Cohen’s d
Positive affect	21.19 ± 2.38	26.70 ± 1.97	−13.25	< 0.001	0.79
Negative affect	29.15 ± 2.51	22.05 ± 2.44	15.03	< 0.001	0.82

Data are presented as Mean ± Standard Deviation.

### 3.3 Comparison of students’ emotion regulation strategies

As presented in Table 3, independent-samples t-tests revealed significant differences between the two groups in both emotion regulation strategies following the intervention. The experimental group demonstrated significantly greater use of cognitive reappraisal and significantly less reliance on expressive suppression compared to the control group, with moderate to large effect sizes.

**Table 3:** Comparison of ERQ scores between groups.

Dimension	Control group (*n* = 54)	Experimental group (*n* = 56)	*t*	*p*	Cohen’s d
Cognitive reappraisal	29.31 ± 2.79	34.11 ± 2.61	−9.30	<0.001	0.66
Expressive suppression	15.89 ± 1.93	10.63 ± 2.36	12.82	<0.001	0.76

Data are presented as mean ± standard deviation.

### 3.4 Comparison of students’ empathic abilities

As shown in Table 4, independent-samples *t*-tests revealed significant differences between the two groups across all dimensions and the total score of the JSE-HPS following the intervention. The experimental group achieved significantly higher scores on perspective-taking, compassionate care, standing in the patient’s shoes, and total empathy, with effect sizes ranging from moderate to large.

**Table 4:** Comparison of JSE-HPS scores between groups.

Dimension	Control group (*n* = 54)	Experimental group (*n* = 56)	*t*	*p*	Cohen’s d
Perspective-taking	61.48 ± 3.38	67.48 ± 3.43	−9.24	<0.001	0.65
Compassionate care	37.17 ± 3.01	41.48 ± 3.43	−7.00	<0.001	0.55
Standing in patient’s shoes	8.65 ± 1.51	11.66 ± 1.53	−10.41	<0.001	0.70
Total empathy score	107.30 ± 7.87	120.63 ± 8.36	−8.81	<0.001	0.63

Data are presented as mean ± standard deviation.

### 3.5 Comparison of students’ attitudes towards communication skills learning

As presented in Table 5, significant differences were observed between the two groups across all CSAS measures. The experimental group scored significantly higher on positive attitudes and total score, both with large effect sizes. For negative attitudes, the Mann–Whitney *U* test revealed that the experimental group’s mean rank was significantly lower than that of the control group.

**Table 5:** Comparison of CSAS scores between groups.

Dimension	Control group (*n* = 54)	Experimental group (*n* = 56)	Test statistic	*p*	Cohen’s d/r
Positive attitudes	50.06 ± 2.01	58.39 ± 2.43	*t* = −19.55	<0.001	0.89
Negative attitudes^a^	79.02^b^	32.82^b^	*U* = 242.000 *Z* = −7.610	<0.001	0.73

Data for positive attitudes are presented as mean ± standard deviation.

a For the CSAS negative attitudes subscale, which violated parametric assumptions, the Mann–Whitney *U* test was used; data are presented as mean rank.

b Mean rank.

### 3.6 Comparison of students’ nursing ethical decision-making competencies

As shown in Table 6, independent-samples t-tests revealed significant differences between the two groups across all JAND measures following the intervention. The experimental group scored significantly higher on moral reasoning, moral behavior, and total score, with moderate to large effect sizes.

**Table 6:** Comparison of JAND scores between groups.

Dimension	Control group (*n* = 54)	Experimental group (*n* = 56)	*t*	*p*	Cohen’s d
Moral reasoning	153.06 ± 8.44	168.14 ± 8.50	−9.34	<0.001	0.66
Moral behavior	148.17 ± 8.31	158.14 ± 8.50	−6.38	<0.001	0.50
Total score	301.22 ± 16.75	326.29 ± 17.01	−7.98	<0.001	0.59

Data are presented as mean ± standard deviation.

### 3.7 Participant safety and monitoring

Throughout the study period, no participants exhibited distress requiring referral to the university’s counseling center, indicating that the intervention was conducted within a psychologically safe framework.

## 4 Discussion

Previous research on SEL has predominantly focused on its role in child and adolescent development (23–25), or explored its application in general education and some health professions training (9). There remains a paucity of interventional research evidence concerning the systematic integration of SEL into nursing curricula and the evaluation of its effects, particularly for preparing trainees for this emotionally demanding profession. This study employed a quasi-experimental design with a control group to systematically assess the pedagogical outcomes of integrating SEL into the *Community Nursing* course. The results indicate that students in the experimental group demonstrated significantly higher levels of positive affect, greater use of cognitive reappraisal for emotion regulation, stronger empathic abilities across all dimensions, more positive attitudes towards learning communication skills, and enhanced nursing ethical decision-making competencies compared to the control group. Conversely, they reported significantly lower levels of negative affect, less reliance on expressive suppression, and fewer negative attitudes towards communication skills learning. These findings provide initial empirical evidence that systematically incorporating SEL into the *Community Nursing* curriculum may enhance nursing students’ socio-emotional competencies and improve their psychological states relevant to professional adaptation, all while ensuring the delivery of core professional knowledge.

### 4.1 Mechanisms of SEL-integrated instruction on emotional experience and regulation

This study found that students in the experimental group reported higher levels of positive affect, lower levels of negative affect, and a greater propensity to use cognitive reappraisal for emotion regulation. This outcome can be understood through Gross’s process model of emotion regulation, which identifies cognitive reappraisal as an antecedent-focused strategy that operates by reinterpreting an emotion-eliciting situation before the full emotional response unfolds (26). The structured reflective activities in our SEL-integrated instruction were designed to practice this strategy by guiding students to re-evaluate challenging simulated scenarios, thereby cultivating a habitual reappraisal response. In practice, community nurses often work independently, managing unexpected emotional conflicts without immediate support, which demands strong emotion regulation capacity. Our intervention, by providing a psychologically safe environment and structured reflective support, helped students accumulate these critical psychological resources. Consequently, they demonstrated greater resilience when confronting emotional challenges. This finding is consistent with Gidalevich et al. (27), whose SEL intervention also led to significant gains in preservice teachers’ cognitive reappraisal, providing direct empirical support for the efficacy of such integrated training.

### 4.2 The unique value of SEL-integrated instruction in cultivating empathy

Students in the experimental group showed significant superiority over the control group across all three dimensions: perspective-taking, compassionate care, and standing in the patient’s shoes. This aligns with the family-centered service philosophy inherent to *Community Nursing* and provides initial support for the potential of SEL-integrated instruction to foster this competency.

Family- and community-centered practice requires nurses to navigate the needs and perspectives of multiple clients within complex interpersonal networks. The community nursing context is characterized by high complexity and interactivity, demanding that nurses accurately understand the perspectives and feelings of their clients. In this study, the experimental group engaged in activities such as simulated home visits to families from diverse cultural backgrounds, navigating conflicts arising from differing health beliefs. The geriatric nursing module incorporated “life value reflection and communication” exercises, prompting students to examine their own attitudes toward aging. These activities required students to enter and operate within multi-member, multi-perspective interpersonal networks for emotional cognition and communication practice. Through repeated exercises in cross-cultural perspective-taking, structured empathic dialogues with standardized patients portraying early-stage dementia, and life story interviews, students consistently practiced empathic responses. This translation of experiential practice into measurable empathy gains can be explained by Kolb’s experiential learning theory (16). The cycle of concrete experience (role-play) followed by reflective observation (debriefing) helps consolidate empathic responding from a situational behavior into a more stable internal competency, as captured by the JSE-HPS.

Previous research, where empathy communication training based on the empathic opportunity-response model was administered to nursing interns, demonstrated significant post-training improvements in both clinical communication and empathic abilities (28, 29). This underscores the distinct advantage of experiential teaching strategies in cultivating emotional competencies. Building upon this foundation, the present study elevates empathy training from a singular focus on communication skills to a systematic developmental program integrated within the framework of the five core SEL competencies. Furthermore, it designs more contextually embedded instructional activities tailored specifically to community nursing scenarios, thereby creating a tighter linkage between empathy cultivation and the demands of professional practice.

### 4.3 Impact on attitudes toward communication skills learning

Effective community health education relies on strong communication skills, which must be adapted to diverse client contexts. The present study found that students in the experimental group exhibited significantly more positive attitudes and less negative attitudes toward learning communication skills compared to the control group.

This shift may be associated with changes in the instructional approach. Instructors in the experimental group, having undergone specialized training, transitioned from traditional knowledge transmitters to learning facilitators and emotional catalysts. It is plausible that they provided constructive feedback on students’ emotional expressions and communicative behaviors, moving beyond merely evaluating factual correctness. This supportive learning environment could help reduce students’ anxiety and resistance, potentially enhancing their learning motivation (30, 31). The cultivation of communication competence should be integrated throughout the nursing curriculum and deeply intertwined with professional content. Simply lecturing on communication theory or conducting isolated skills training is insufficient to motivate students (32). However, when communication training is closely integrated with authentic community nursing scenarios, students personally experience how effective communication builds patient trust and promotes health behavior change. This naturally strengthens their positive attitudes toward learning these skills.

### 4.4 Impact of SEL-integrated instruction on ethical decision-making competency

Community nursing practice frequently involves navigating ethical dilemmas under resource constraints, necessitating responsible decisions that align with professional ethics (33, 34). In this study, the experimental group demonstrated significantly higher scores than the control group in moral reasoning, moral behavior, and overall ethical decision-making. These findings may have implications for both theory and practice.

The instructional design for the experimental group may have contributed to this outcome through activities such as collaborative project planning and ethical deliberation debates. These tasks required students to integrate core values like justice and harmony into their professional decision-making practice. In the community nursing context, justice relates to equitable care access, while harmony involves balancing individual needs with family and community dynamics. The JAND instrument, applied effectively in similar educational contexts (35), measures the ability to apply such ethical principles. Thus, when faced with complex dilemmas, students in the experimental group appeared better equipped to formulate decisions that adhered to professional standards while incorporating a humanistic perspective.

### 4.5 Implications for nursing education reform

The findings of this study, which demonstrated significant improvements in students’ emotion regulation, empathy, attitudes toward communication, and ethical decision-making, point to the potential for socio-emotional competencies to be cultivated through intentional pedagogical design. Community nursing practice, with its family-centered focus, reliance on health education, and operation within resource constraints, places particular demands on abilities that align with the core domains of SE. Thus, integrating SEL into the Community Nursing curriculum may be viewed not simply as an adjunct to humanistic education but as a response to the intrinsic demands of the course. Nursing education reform could consider these unique requirements and explore ways to integrate SEL objectives with core professional content.

Although this study focused on community nursing education, the core competencies addressed are foundational to nursing and health professions education broadly. The SEL framework may hold relevance for other contexts, such as acute care, mental health nursing, or interprofessional training, where these relational and self-regulation skills are critically important. Future research should investigate adapting and evaluating SEL-informed approaches in these diverse settings.

### 4.6 Limitations

Several limitations merit consideration. First, the modest sample from a single institution limits statistical power and generalizability. Second, reliance solely on quantitative scales precluded in-depth exploration of participants’ subjective experiences. Third, the quasi-experimental design assigned intact classes without randomization constrains causal inference. Fourth, the lack of pre-intervention measurements on the primary outcome variables limits the ability to fully attribute post-intervention differences to the intervention. Fifth, the potential for contamination between groups could not be fully ruled out. Finally, social desirability bias may have influenced self-reported responses.

## 5 Conclusion

The results suggest that the potential benefits of SEL-integrated instruction may stem from mechanisms such as contextualized simulation, structured reflection, and supportive feedback. These mechanisms appear to foster the transition of socio-emotional competencies from explicit learning to internalized application. To advance nursing education, it would be valuable to consider the systematic design of emotional competence cultivation within professional courses, seamlessly integrating SEL goals with disciplinary content. This could involve strengthening the central role of guided reflection in the teaching process, enhancing instructors’ training in emotional facilitation, and providing students with ample opportunities to practice emotional and relational skills within psychologically safe learning environments.

## Data Availability

The raw data supporting the conclusions of this article will be made available by the authors, without undue reservation.
